# An Improved Method for Monitoring Multiscale Plant Species Diversity of Alpine Grassland Using UAV: A Case Study in the Source Region of the Yellow River, China

**DOI:** 10.3389/fpls.2022.905715

**Published:** 2022-06-09

**Authors:** Yi Sun, Yaxin Yuan, Yifei Luo, Wenxiang Ji, Qingyao Bian, Zequn Zhu, Jingru Wang, Yu Qin, Xiong Zhao He, Meng Li, Shuhua Yi

**Affiliations:** ^1^School of Geographic Science, Institute of Fragile Eco-Environment, Nantong University, Nantong, China; ^2^State Key Laboratory of Cryospheric Sciences, Northwest Institute of Eco-Environment and Resources, Chinese Academy of Sciences, Lanzhou, China; ^3^School of Agriculture and Environment, College of Science, Massey University, Palmerston North, New Zealand

**Keywords:** species diversity, diversity monitoring, unmanned aerial vehicle, FragMAP, multiscale diversity

## Abstract

Plant species diversity (PSD) is essential in evaluating the function and developing the management and conservation strategies of grassland. However, over a large region, an efficient and high precision method to monitor multiscale PSD (α-, β-, and γ-diversity) is lacking. In this study, we proposed and improved an unmanned aerial vehicle (UAV)-based PSD monitoring method (UAV_B_) and tested the feasibility, and meanwhile, explored the potential relationship between multiscale PSD and precipitation on the alpine grassland of the source region of the Yellow River (SRYR), China. Our findings showed that: (1) UAV_B_ was more representative (larger monitoring areas and more species identified with higher α- and γ-diversity) than the traditional ground-based monitoring method, though a few specific species (small in size) were difficult to identify; (2) UAV_B_ is suitable for monitoring the multiscale PSD over a large region (the SRYR in this study), and the improvement by weighing the dominance of species improved the precision of α-diversity (higher *R*^2^ and lower *P* values of the linear regressions); and (3) the species diversity indices (α- and β-diversity) increased first and then they tended to be stable with the increase of precipitation in SRYR. These findings conclude that UAV_B_ is suitable for monitoring multiscale PSD of an alpine grassland community over a large region, which will be useful for revealing the relationship of diversity–function, and helpful for conservation and sustainable management of the alpine grassland.

## Introduction

Plant species diversity (PSD) describes vegetation community structures and plays a critical role in ecosystem multifunction and conservation of grassland (Lu and He, 2017; Moorsel et al., 2018; Pires et al., 2018; Yamamura et al., 2021). Investigating the spatial and temporal variations of species diversity in natural real-world communities provides essential information for understanding ecosystem multifunction and evolutionary processes, and developing reasonable conservation and management strategies of grassland (Hodkinson, 2005; Genung et al., 2020; Michaels et al., 2021).

To depict vegetation community structures and biotic heterogeneity at multiple scales, α-, β-, and γ-diversity are implemented (Bello et al., 2006; Sun et al., 2018). α-diversity indices indicate the number and abundance of species within local communities, and the indices generally refer to the number of species present (e.g., species richness), heterogeneity or diversity (e.g., Shannon index and Simpson index) (Spellerberg and Fedor, 2003; Wesuls et al., 2013), and evenness of species abundances (e.g., Pielou's *J* index) (Karen et al., 2004). β-diversity is defined as the variation in species composition among communities, which is closely related to the regional species pool and large-scale patterns of species richness (e.g., Sørensen index and Cody index) (Whittaker, 1960). γ-diversity is usually defined as the total number of species present in a region (Mori et al., 2018). In the context of global change (Laender et al., 2016), research on biodiversity is now moving toward evaluating the potential effects in real-world ecosystems, advancing the model systems that focus on experimental and manipulative results (Soliveres et al., 2016; Mori, 2018). Given the environmental heterogeneity of real-world ecosystems, multiscale monitoring could play a central role in understanding how these naturally diverse and fluctuating communities are organized, and how such processes influence the functioning of ecosystems. However, the variation in the identities and abundances of species among local assemblages (i.e., β-diversity) has received much less attention (Mori et al., 2018), and the most important reason is that, at a large scale, the efficient and high precision method that is applied to monitor the multiscale PSD is still lacking to date.

The heterogeneous distribution of species across space and time calls for a more dynamic appraisal of diversity (Mori et al., 2018). A variety of methods, for example, the MODIS, Landsat, and Quickbird imagery have been developed and applied to investigate PSD (Langley et al., 2001; Hall et al., 2009; Huang et al., 2009). However, those methods based on space-borne imagery cannot resolve grassland species composition at a species level (millimeter level) because species in grasslands are typically small in size and highly mixed (Lu and He, 2017; Sun et al., 2018). Therefore, the traditional ground-based survey method (quadrat or belt method) is still employed in the studies of species composition and diversity of grassland (Sun et al., 2015; Yamamura et al., 2021). Nevertheless, the traditional ground-based methods (TSM_Q_) have several drawbacks. First, they are time-and money-consuming and labor-intensive (Ge et al., 2018; Sun et al., 2018), making it hard to monitor PSD over a large region repeatedly, which is essential in the studies of multiple diversity. Second, the results are not comparable among different spatial and temporal sampling schemes (Chillo et al., 2015). Third, monitoring the dynamic of PSD at fixed points is challenging due to destructive sampling.

An unmanned aerial vehicle (UAV) has been applied to study the species composition at the species level in recent years (Baena et al., 2017; Lu and He, 2017). Nevertheless, only specific species are focused on, and the results are not comparable to traditional monitoring and analysis methods (Lu and He, 2017; Sun et al., 2018). Sun et al. (2018) proposed a UAV-based species composition monitoring method (UAV_B_) that could monitor species composition dynamics at fixed points and is comparable to the traditional ground-based monitoring methods. The feasibility of UAV_B_ has been tested in some alpine regions, focusing on the α-diversity at a local scale (Qin et al., 2020; Wei et al., 2021). However, this method could not consider differences among species in the subplot (one of the 16 aerial photographs), i.e., it is based on the presence of each species that may cause the results to differ from those collected by the ground-based method (Sun et al., 2018). In the early 1960's, Mannetje and Haydock (1963) proposed a dry-weight-rank (DWR) method to estimate the botanical composition based on the dry weight of each species, which provides a potential way to solve the heterogeneity in a subplot and improves the estimate precision by weighting dominance of species.

The Qinghai-Tibetan Plateau (QTP) is referred to as the “third pole” because of its importance as an ecoregion. The source region of the Yellow River (SRYR) is located in the northeastern QTP (Chu et al., 2016). The alpine grassland in SRYR is essential for animal husbandry and ecological security of China, while it is also a fragile ecosystem and is sensitive to anthropogenic disturbance and climate change (Li et al., 2016; Ge et al., 2018). Based on the investigations over time at a small scale or across space for a short duration and model simulation methods, it is found that the precipitation is confirmed to be a principal driver that promotes the species diversity of alpine grassland (Ma et al., 2010; Kreyling et al., 2017; Li et al., 2020). In this study, we investigated the species diversity in the sites with different annual precipitation by UAV-based and ground-based methods; furthermore, we explored the relationship between PSD and precipitation in SRYR. The specific objectives were to: (1) test the feasibility of UAV_B_ for investigating vegetation species diversity at multiple scales, (2) improve the precision of the UAV_B_ by weighing the dominance of species in subplot (the single aerial photograph), and (3) explore the tendency of species diversity along the precipitation gradient in SRYR. Our results could have significant implications on land-use policies and socio-ecological sustainability practices on QTP.

## Materials and Methods

### Study Sites

**Figure 1:**
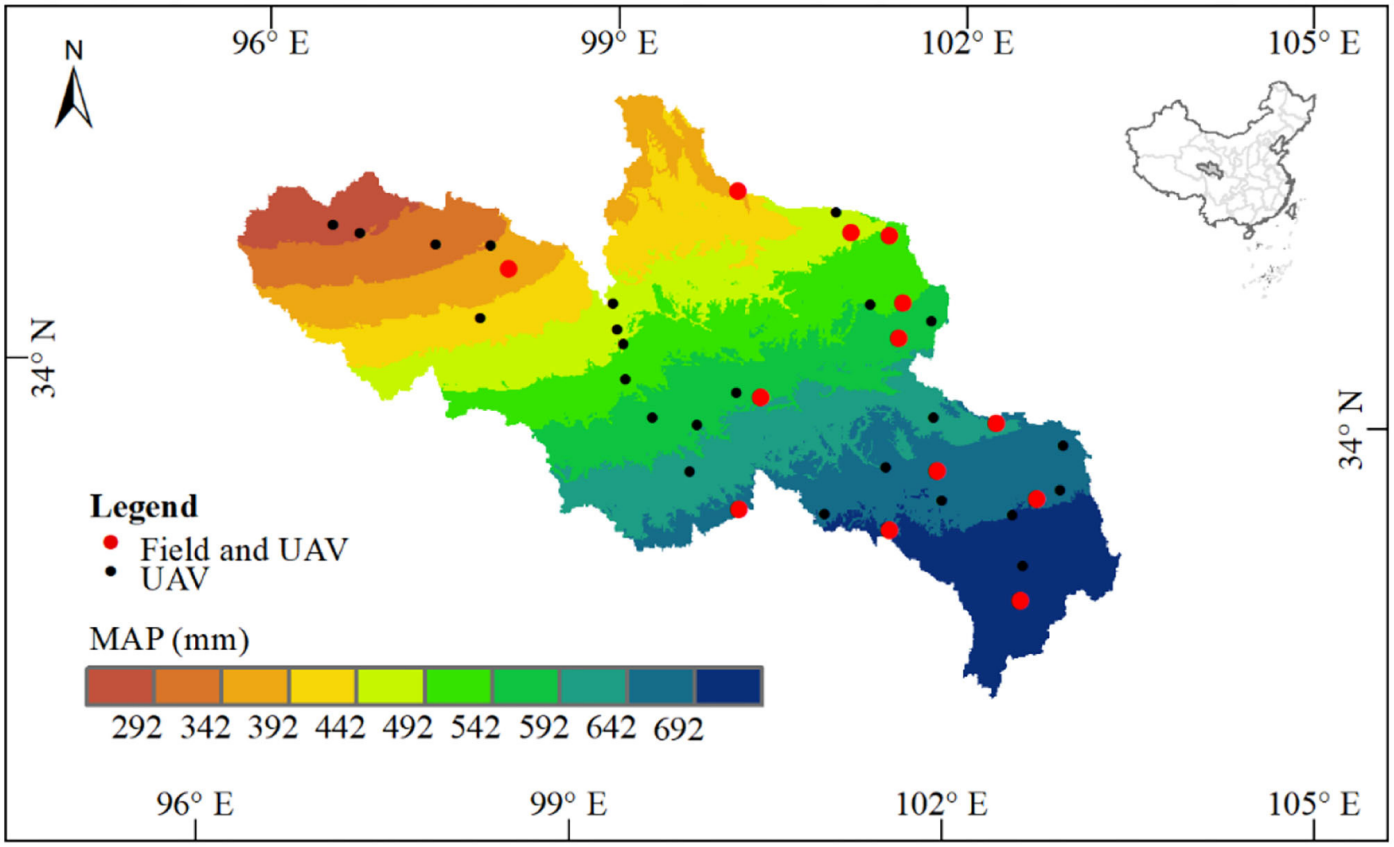
Spatial annual precipitation (mean of 2000–2017) and the location of sampling sites among the source region of the Yellow River on the Qinghai-Tibetan Plateau.

This study was conducted in SRYR (N33°02′58″-36°07′43″, E95°53′47″-102°15′22″; Figure 1). The SRYR covers an area of ~105,190 km^2^, featured with high elevation (~ 3,800 m a.s.l.), low annual temperatures, large diurnal temperature differences, seasonal precipitation extremes, intense evaporation, and strong solar radiation (Ge et al., 2018). In the SRYR, the annual temperature is between −4 and 2°C, the annual precipitation is ~420 mm, and mostly from June to September (Li et al., 2016). Grasslands play a vital role in protecting the ecological environment and preventing land degradation in SRYR (Feng et al., 2005; Zhou et al., 2005). The soils are mainly alpine meadow and steppe soils (Yu et al., 2014).

### Field Sampling and Data Collection

Between July and August of 2018, we conducted field sampling at 37 sites in SRYR using the UAV_B_ method (see below for more details). The field sampling and data collection contained four contents: (1) taking aerial photographs by the UAV at 2 m height, (2) sampling on the ground to test the feasibility of the UAV for vegetation species diversity investigation, (3) identifying vegetation species from each photograph, and (4) collecting the precipitation data. Detailed information is listed in the following sections.

#### UAV Aerial Photographing

A lightweight four-wheel drone equipped with an omnidirectional sensing system and autopilot system was used in this study—Mavic 2 Pro (DJI Innovation Company, China). Vertical and horizontal accuracies of this drone are ± 0.5 and ± 1.5 m, respectively. The maximum flying height could reach 6,000 m above sea level, and the hovering endurance is 30 min. A standard built-in 12-megapixel RGB camera (DJI Innovation Company, China) is equipped, and the field vision is 83° at 24 mm focus. The longitude and latitude of the central position are recorded in each aerial photograph. The terrain-following function of Mavic Pro maintains similar areas in each photograph.

**Figure 2:**
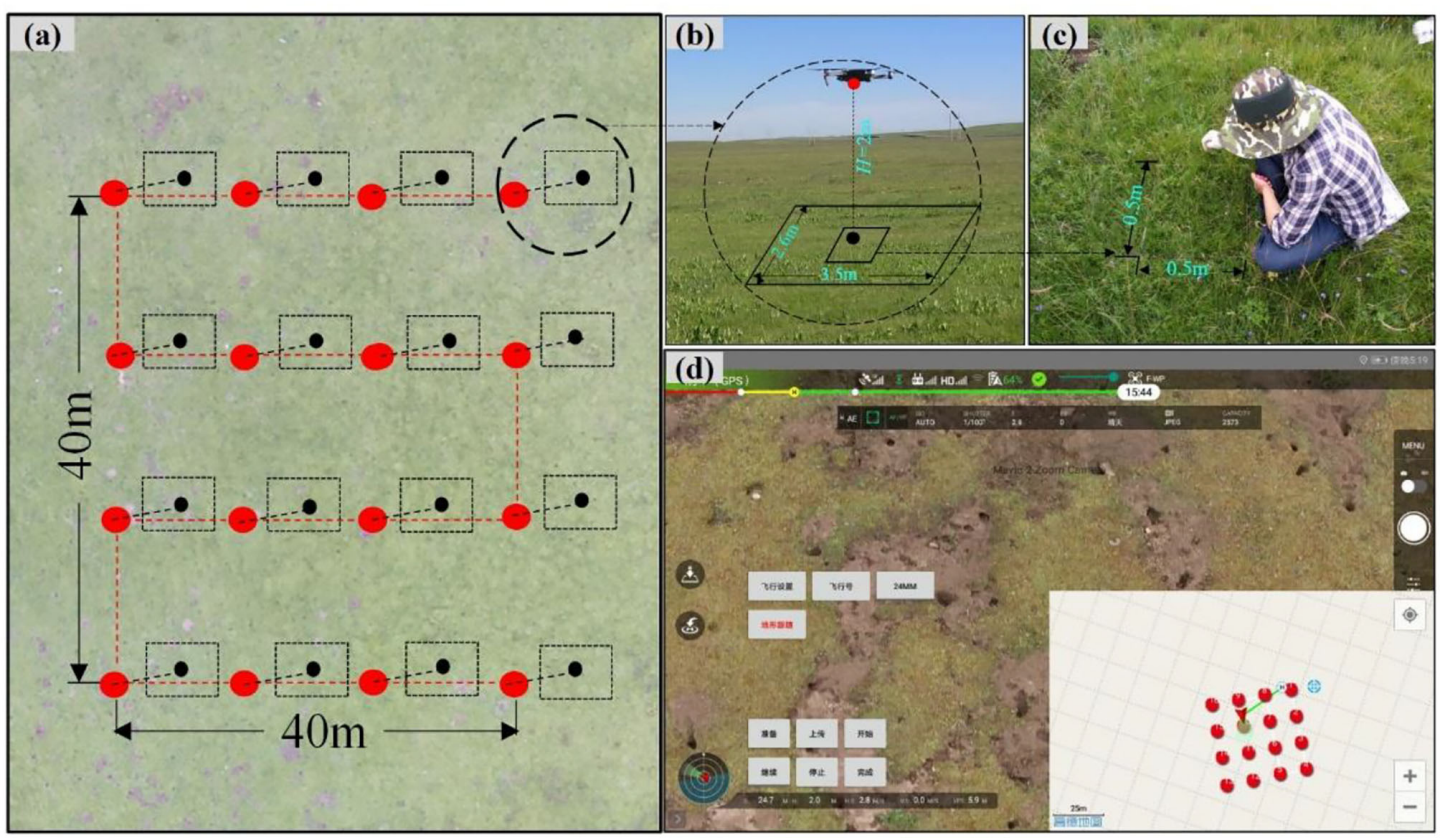
Field sampling method: **(a)** waypoints (large red dots), flight trajectory (red dashed line), and area of aerial photograph (black frame with the center represented by black dots); **(b)** aerial photograph taken by MAVIC Pro at the height of 2 m; **(c)** traditional ground-based measurement (3 quadrats selected randomly among 16 sampling sites); and **(d)** real-time sampling along the “Belt” trajectory of FragMAP system.

Vegetation species were investigated using the traditional ground-based method (TSM_Q_) and UAV_B_ methods to evaluate species diversity, and the feasibility of the UAV_B_ was tested on a large scale. Briefly, on each site, one Belt route (40 m × 40 m, Figures 2a,d) was set up, and the UAV was auto-piloted to the 16 preset way points to take aerial photographs at 2 m vertically using Fragmentation Monitoring and Analysis with aerial Photography (FragMAP) (Yi, 2017). The flight speed was preset to 4 ms^−1^, and the resolution of each pixel on the ground was ~0.09 cm, which is suitable for identifying each species accurately (Sun et al., 2018; Qin et al., 2020). The UAV hovered for 3 s over each way point and took photographs within 1 s. Overall, 592 aerial photographs were taken to identify vegetation species visually.

#### Sampling on Ground

Thirteen of 37 sampling sites were randomly selected to sample species composition using TSM_Q_. In each Belt route, three quadrats (0.5 × 0.5 m) were placed in the way points 6, 7, and 11 before the UAV arrived (Figures 2b,c). After aerial photographing, all vegetation species found within each quadrat were identified visually. Then, each species was clipped at the ground level, dried (oven-dried at 65°C for 48 h) and weighed in a laboratory, and average values were used in statistical analyses.

#### Extracting Information From Aerial Photographs

We were aware that there was a little bit of distortion around the margin of aerial photographs, even though we took them vertically down. However, it was reasonable to extract data directly (no referencing and rectification) from the aerial photographs, as they were collected using the same method and using the same type of UAV; therefore, the processes of identifying species meet the study accuracy requirements (Sun et al., 2018; Qin et al., 2020). In this study, we identified and recorded all the species presented within each aerial photograph, and calculated the focused diversity indices based on the frequency of species (Sun et al., 2018). Furthermore, we weighted the dominance of each species based on the visually estimated dominance, which followed the ideas of Mannetje and Haydock (1963), based on the data statistical method. The detailed method about dominance weighting is listed in Diversity Indices.

#### Collecting Precipitation Data

Based on the daily values of precipitation from 2000 to 2017 of all the stations on QTP of China [the China Meteorological Data Service Center (CMDC) (http://data.cma.cn/en)], we calculated the monthly sums for precipitation and followed the instruction of Chen et al. (2014) to interpolate the monthly weather data into raster surfaces with a spatial resolution of 1 × 1 km with ANUSPLIN 4.3 (Hutchinson, 2004; Li et al., 2020).

### Diversity Indices

Usually, several measures can be combined for an in-depth description of vegetation diversity (Bonham, 2013). To assess the feasibility of using the UAV_B_ methods to quantify PSD of grassland at multiple scales in the SRYR, four common indices, the species richness, Shannon index, Simpson index, and Pielou's *J* index, were used to indicate the community assembly at a local scale, with all four indices used for α-diversity (Spellerberg and Fedor, 2003; Karen et al., 2004); at a regional scale, the Sørensen index and Cody index were used to indicate variation in species composition and substitution rate among communities along specific environmental gradients (Mori et al., 2018); at a regional or larger scale, the total number of species is used for describing PSD in a specific region (i.e., -diversity) (Whittaker, 1960).

In this study, species richness was calculated using Eq. (1) (Bonham, 2013):


(1) $$\begin{array}{l} N\;=\text{ number of species presence of unit area} \end{array}$$

where *N* is the total number of species per sampling site (16 aerial photographs, UAV_B_) or quadrat (3 quadrats, TSM_Q_).

The Shannon (*H*) and Simpson (*D*) diversity indices were calculated using Eqs. (2) and (3), respectively (Spellerberg and Fedor, 2003; Wesuls et al., 2013):


(2) $$\begin{array}{l} H\;=\;-\sum_{\text{n}\;=\;1}^{16}p_{\text{i}}\ln\;p_{\text{i}} \end{array}$$

(3) $$\begin{array}{l} D\;=\;1-\sum_{n\;=\;1}^{16}p_{i}^{2} \end{array}$$

where *p*_*i*_ is the proportion [i.e., (biomass proportion + density proportion)/2] of *i* species in each quadrat (TSM_Q_), frequency of species *i* in 16 photographs of each sampling point (UAV_B_), or dominance of species *i* in 16 photographs of each sampling site (UAV_BD_, see below for more details).

Pielou's *J* index was calculated using Eq. (4) (Karen et al., 2004):


(4) $$\begin{array}{l} P\;=\;H/lnN \end{array}$$

Given the heterogeneity among the subplots, we improved the method applied to calculate the commonly used diversity indices by adding weight dominance of species, i.e., we weighted the dominance species as 3, the subdominant species as 2, and all the other species presence as 1. The number of dominant and subdominant species was set within 0–2 based on the dominance of each species based on aerial photographs (see the potential possible cases in Supplementary Table S2).

For the β-diversity, the Sørensen (Sβ) and Cody (Cβ) diversity indices were calculated using Eqs. (5) and (6), respectively (Whittaker, 1972):


(5) $$\begin{array}{l} S\beta \;=\;\frac{2c}{a\;+\;b} \end{array}$$

(6) $$\begin{array}{l} C\beta \;=\;\frac{g\left(H\right)\;+\;l\left(H\right)}{2}\;=\;\frac{a\;+\;b\;-\;2c}{2} \end{array}$$

where *a* and *b* are the species numbers in two sampling units (the quadrats or Belt route), *c* is the common species number in the two sampling units. *g*(*H*) and *l*(*H*) represent the species numbers increase or decrease along the habitat gradients (*H*), respectively. In this study, 8 (13 sampling sites, no sampling sites around the areas with lower precipitation, Supplementary Figure S1) and 10 groups (37 sampling sites; Figure 1) were separated with a 50-mm interval along the precipitation. We compared the β-diversity of adjacent groups (based on the mean values of each group) to depict differentiation characteristics of alpine grassland communities in SRYR. The total species number identified by different methods represented the γ-diversity of the SRYR.

### Statistical Analysis

A goodness-of-fit test (Shapiro-Wilk test and univariate procedure) was used to test the normality of data. The coefficient of determination (*R*^2^) and its *P* values were used to evaluate different sampling methods for α- and β-diversity. Analyses of indices were performed utilizing the VEGAN package in R (R Core Team, 2013). To select the final regression models, which indicated the effect of precipitation on species composition, the likelihood ratio test was used to compare the simple linear regression and polynomial regression models (ggplot2 package in R). The statistical analyses were performed with R version 4.0.5.

## Results

### The Improvement of UAV_B_ in Monitoring Multiscale Diversity

The UAV_B_ monitored more species (*n* = 99) than the TSM_Q_ method (*n* = 92) within the same sampling sites (Supplementary Table S1). Furthermore, 8 species were found in the other 24 sampling sites that were only monitored by the UAV_B_ method (Supplementary Table S1). Altogether, there were 111 species identified in SRYR in this study (Supplementary Table S1). Therefore, the UAV_B_ method is more representative of the γ-diversity at a regional scale (the SRYR in this study).

**Figure 3:**
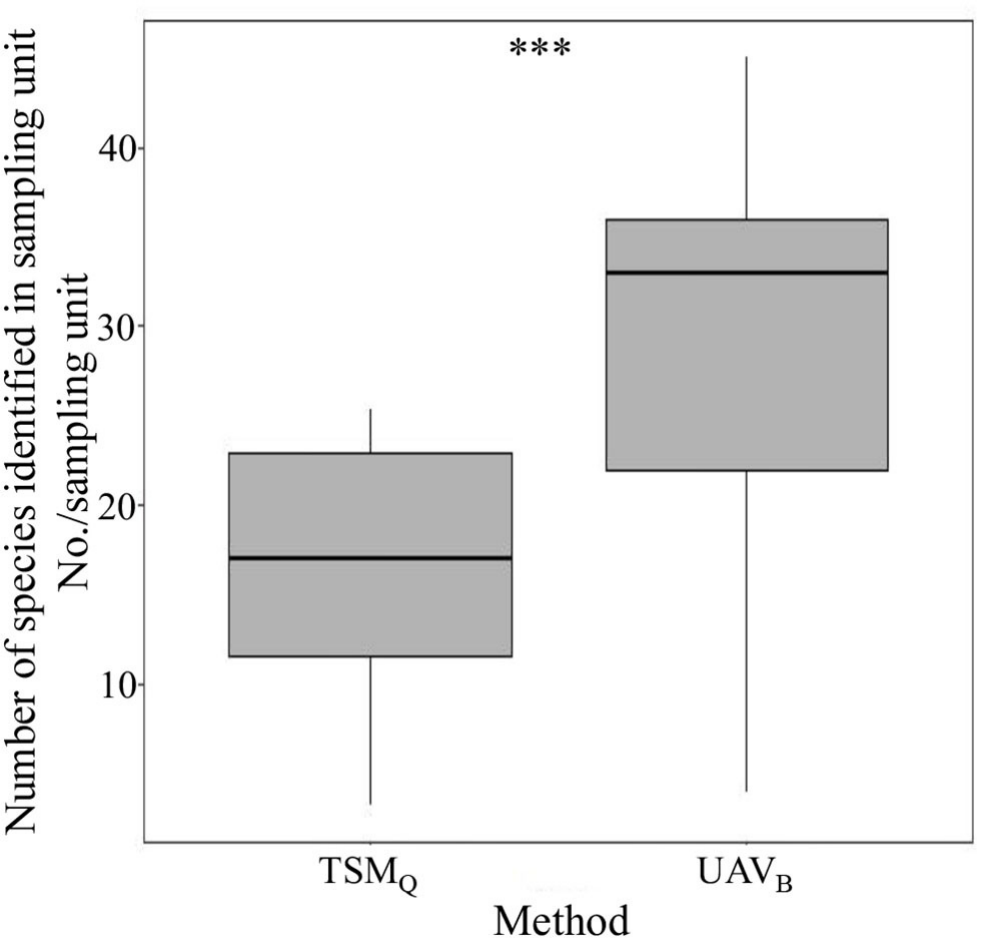
The number of species identified within the sampling unit by the traditional sampling method (TSM_Q_) and the UAV-based method (UAV_B_). ^***^Indicates significant difference at *P* < 0.001 level.

Within sampling units (one Belt route of UAV_B_, or three quadrats of TSM_Q_), the average species number identified by the UAV_B_ method was significantly higher than that identified by TSM_Q_ (*P* < 0.01; Figure 3). There were only four species (i.e., *Arenaria serpyllifolia, Scirpus pumilus, Equisetum arvense*, and *Geranium wilfordii*) that could not be recognized by the UAV_B_ method, while 11 species failed to be recorded by the TSM_Q_ method (Supplementary Table S1). These results indicated that the richness (α-diversity) observed by UAV_B_ is more representative than TSM_Q_ at a local scale.

### Comparisons of Multiscale Diversity Estimated by UAV_B_ and TSM_Q_ Methods

**Figure 4:**
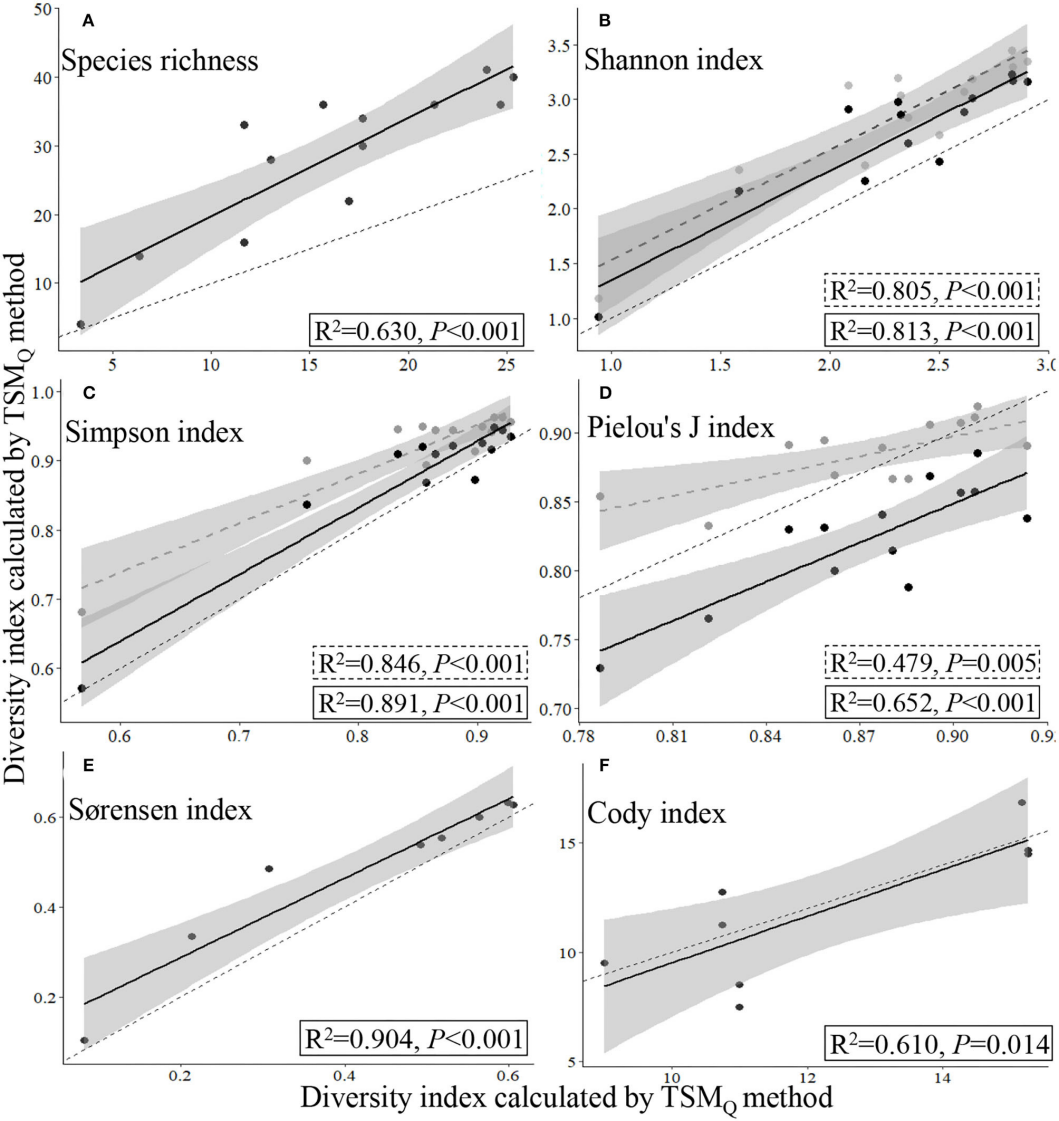
Relationships of α- **(A–D)** and β-diversity indices estimated **(E,F)** by the traditional quadrat-based (TSM_Q_) and UAV-based (UAV_B_) methods. The gray circles and dashed regression lines indicate the presence-based method (UAV_B_), while the black circles and full regression lines indicate the dominance-based method (UAV_BD_) to the α-diversity indices; the thin dashed line represents the 1:1 line.

**Figure 5:**
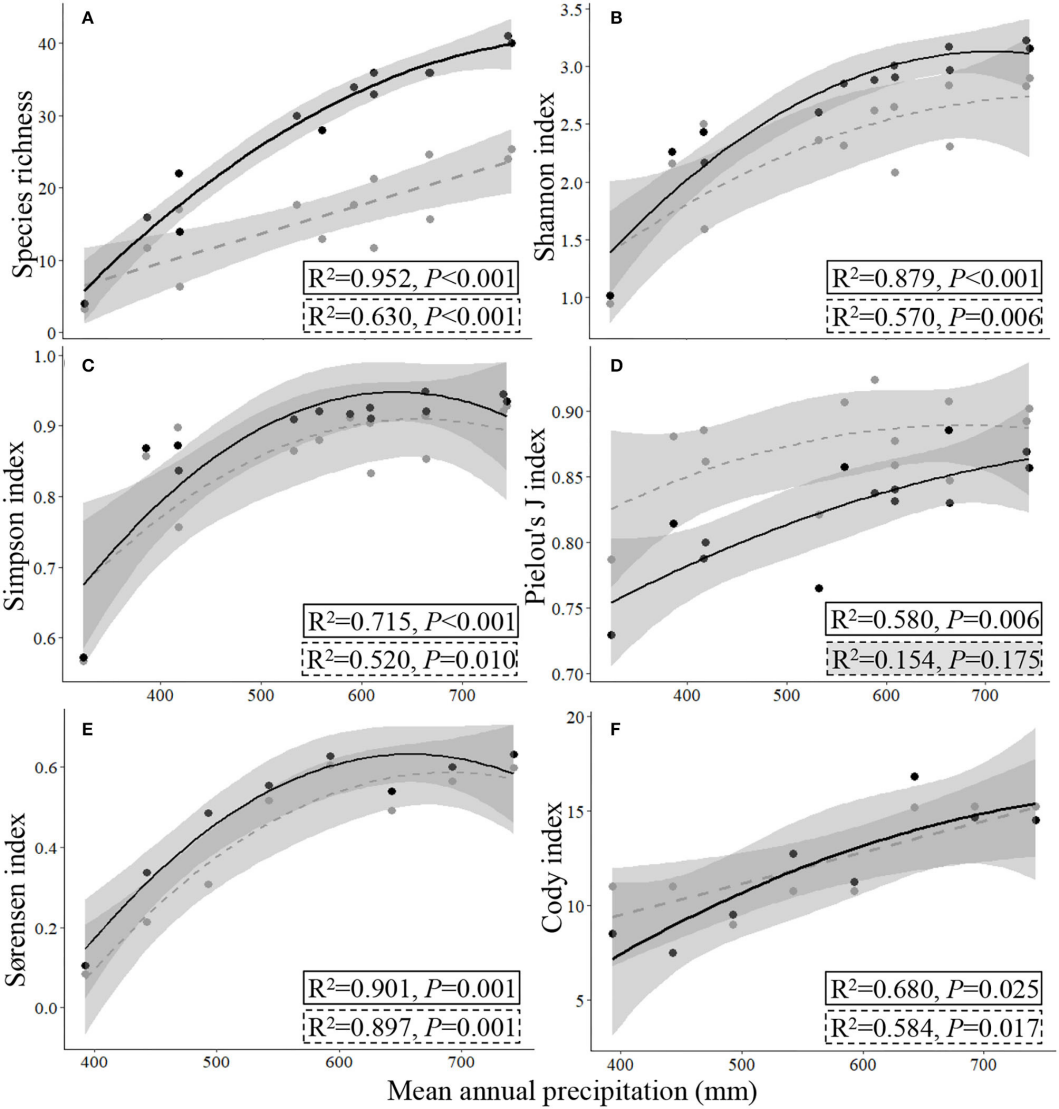
Responses of α- **(A–D)** and β-diversity indices **(E,F)** to mean annual precipitation (2000–2017). The gray circles and dashed regression lines indicate the traditional sampling method (TSMQ), while the black circles and full regression lines indicate the presence-based sampling method (UAV_B_, the improved dominance-based method for A–D, UAV_BD_).

Based on the data collected from the 13 sampling sites by TSM_Q_ and UAV_B_ methods, there were significant linear relationships between the measurements of a given species diversity index (species richness, Shannon index, Simpson index, or Pielou's *J* index for α-diversity, and Sørensen and Cody indices for β-diversity) estimated by the TSM_Q_ and UAV_B_ methods (*P* < 0.01; Figures 4, 5).

The data collected by UAV_B_ could easily and directly supply the data for calculating β- and γ-diversity, as the Sørensen and Cody indices and total species number are calculated based on the presence of the species, refer Eqs. (5) and (6). The regression fitting curves of the Sørensen and Cody indices that were estimated by the TSM_Q_ and UAV_B_ methods were close to the 1:1 line, indicating that the UAV_B_ method could estimate the β-diversity precisely (*R*^2^ = 0.904, *P* < 0.001, and *R*^2^ = 0.610, *P* = 0.014, respectively; Figures 4E,F).

For the α-diversity indices, the method featured with weighting dominance of each species was more precise, i.e., close to the 1:1 line, with higher *R*^2^ and lower *P* values (Figures 4B–D).

### Response of Species Diversity to Precipitation in SRYR

**Figure 6:**
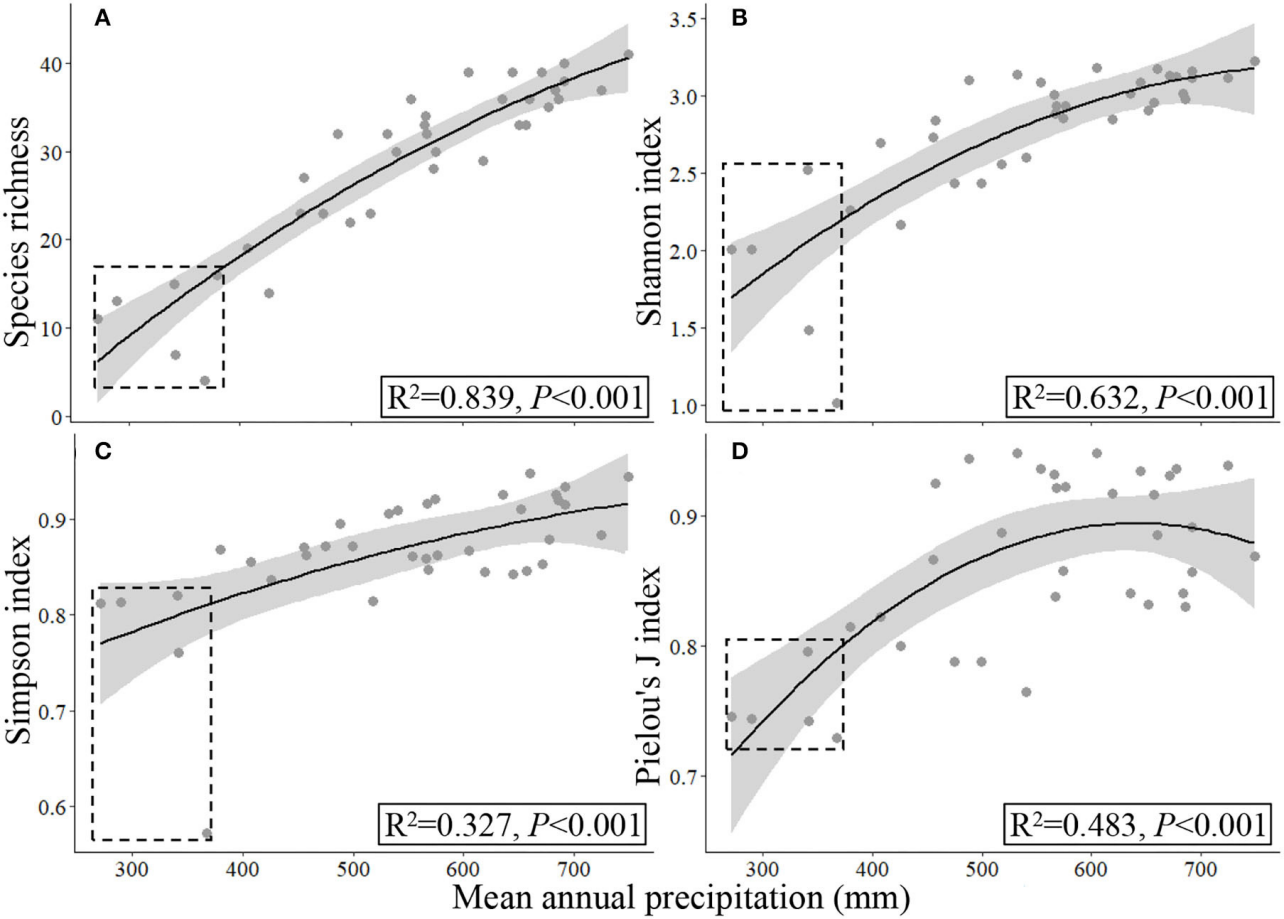
Responses of α-diversity **(A–D)** to mean annual precipitation (2000–2017) based on the data collected by UAV_BD_. The samples sampled in semi-arid regions were marked by dashed rectangles.

**Figure 7:**
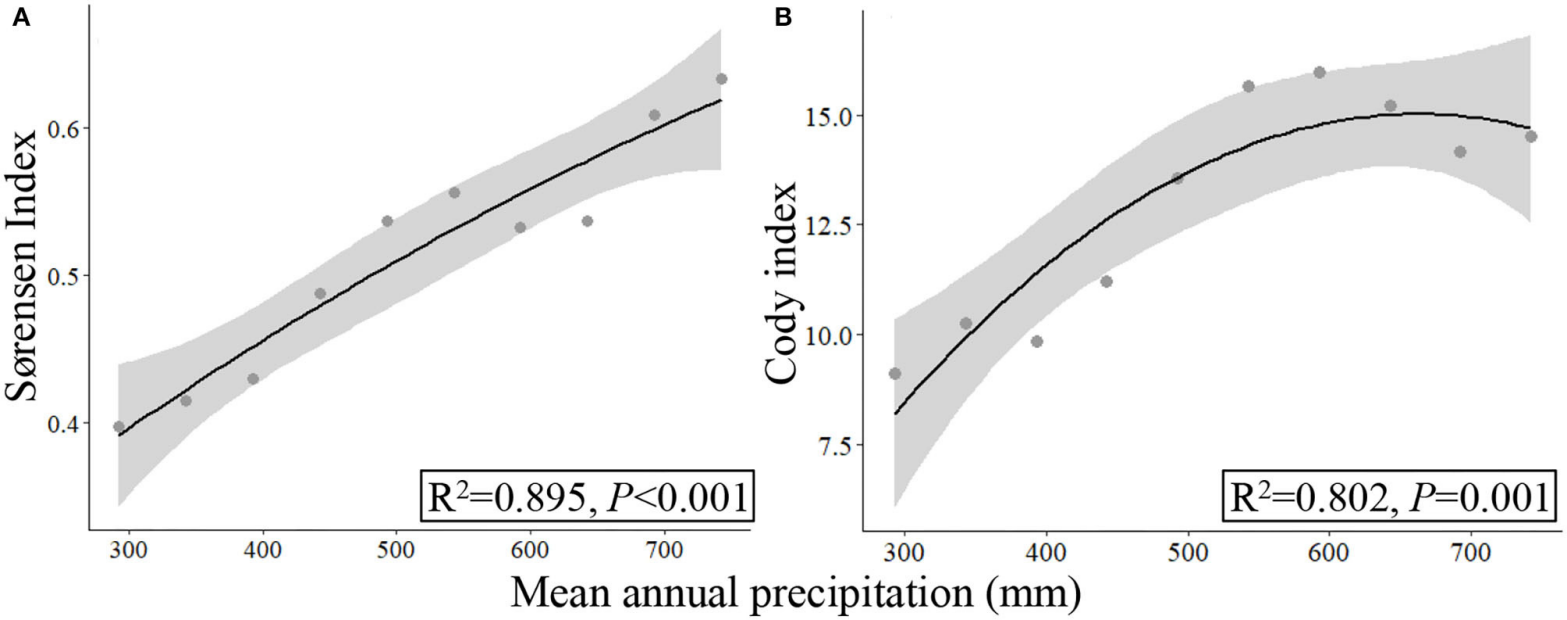
Responses of β-diversity **(A,B)** to mean annual precipitation (2000–2017) based on the data collected by UAV_B_.

In general, with the increase of precipitation, all species diversity indices increased first and then tended to be stable (Figures 5–7). Based on the data collected from the 13 sampling sites by the TSM_Q_ and UAV_B_ methods, the UAV_BD_ method exhibited higher species richness values than the TSM_Q_ method at the same sampling point, and the difference increased with increasing precipitation (Figure 5A). The Shannon index and Simpson index of the UAV_BD_ method had higher *R*^2^ and lower *P* values of fitting curves (Figures 5B,C). Pielou's *J* index calculated by UAV_BD_ exhibited a significant quadratic relationship to precipitation within SRYR, while a non-significant relationship was found based on the data collected by TSM_Q_ (Figure 5D; *P* = 0.175). The Sørensen index and Cody index estimated by UAV_B_ had higher *R*^2^ values of fitting curves, and the Cody index estimated by TSM_Q_ exhibited a linear relationship to the increase of precipitation (*P* < 0.05; Figures 5E,F).

Based on the data collected from all the 37 sampling sites by UAV_B_ methods, the precipitation range extended and the four α- and two β-diversity indices exhibited a significant quadratic relationship in response to the increasing precipitation (Figures 6, 7). However, the coefficients (*R*^2^) were lower, and some data were out of the confidence intervals of fitting curves, especially when they were collected from the semiarid areas (Figures 6A–C).

## Discussion

### Characteristics of the UAV_B_ Method in Multiscale Diversity Monitoring

Representativeness of sampling is greatly influenced by the limited number and size of samples in species diversity monitoring, especially for natural grasslands with high spatial heterogeneity of species features (Wisz et al., 2010; Ge et al., 2018). However, sampling from a large number of quadrats is very time-consuming, labor-intensive, and costly, which is not suitable for sampling surveys over large areas, particularly in a harsh environment such as the QTP featured with high elevation (Ge et al., 2018). In this study, we used the PSD indices of TSM_Q_ [Eqs. (1–6)], but calculated them based on UAV_B_ (for α-, β-, and γ-diversity) or improved UAV_BD_ (only for α-diversity). We found that for each diversity index, a significant linear relationship between the measurements is calculated by UAV_B_ (and improved UAV_BD_) and TSM_Q_ (Figure 4). The results have three implications. First, at a local scale, it could improve the precision of measurement of PSD indices by weighting the species based on dominance, and the UAV_BD_ method can be used to acquire other relevant indices of species diversity of grasslands. Second, at regional and larger scales, the presence-based data collected by UAV_B_ could reveal PSD precisely (Figure 4; Supplementary Table S1). Third, UAV_B_ could cover more species (higher representativeness) and monitor more sampling sites (higher efficiency). Therefore, the UAV_B_ method is more suitable for multiscale heterogeneous grassland species monitoring (Figures 3, 6; Supplementary Table S1), which is crucial in revealing the relationship between species diversity and multifunction of ecosystems (Mori et al., 2018).

Ecological studies often require long-term repeated monitoring to understand the species diversity dynamics in nature (Bonham, 2013; Jorgenson et al., 2015). Meanwhile, species diversity is usually related to the landscape-scale variation (Mori et al., 2018). For example, Karp et al. (2012) demonstrated the scale-dependency of β-diversity in response to the land-use intensification because of a sampling effect. In this study, for each set of waypoints in the field, multiple flights with fixed height (2 m) can be executed at different times to conduct repeated monitoring (Yi, 2017). The UAV_B_ supported by FragMAP makes sure the uniform monitoring standards, avoiding multiscale species diversity variation due to the choice of different metrics (Mori et al., 2018; Zhang et al., 2021). Besides, UAV_B_ can significantly reduce fieldwork time and intensity by reducing individual sampling time and implementing cooperative monitoring (Yi, 2017). Therefore, the UAV_B_ method can be used for multiscale survey/monitoring grassland PSD within a similar phenological period. Meanwhile, the non-intrusive sampling pattern avoided additional disturbance to the sampling sites during the repeated monitoring process (Sun et al., 2018). Furthermore, study areas that are difficult to reach by walking can be easily accessed with a UAV (Floreano and Wood, 2015). Hence, the UAV_B_ method (including the improved UAV_BD_ for estimating α-diversity precisely) can be used for monitoring the dynamics of multiscale species diversity of grassland.

### Species Diversity Along the Precipitation Gradient in Alpine Grassland

High altitude environments are often characterized by low temperatures and short growing seasons, resulting in high plant endemism and biodiversity (Elsen and Tingley, 2015; Verrall et al., 2021). The prevalence of species and changing adaptations along environment gradients provide insights into the potential response of communities to climate change (Hodkinson, 2005). In the near future, the climate is forecasted to be warmer and wetter with shifting precipitation spatiotemporally on QTP (Yang et al., 2014). Researchers have revealed that precipitation is one of the most important factors that regulate species richness and diversity of alpine grassland (Yang et al., 2014; Li et al., 2020), and species diversity indices may increase with increasing precipitation linearly or nonlinearly (Wu et al., 2012). Similarly, we found a quadratic relationship between widely used species diversity indices and precipitation in SRYR (Figures 5–7), implying that with the increase of precipitation, species diversity increased first and then becomes stable to some extent. However, no specific threshold value could be estimated for the species diversity indices along the precipitation gradient (Figures 5–7). One possible reason is that the various biological meanings among the indices, e.g., the α-diversity are usually used for describing local community assembly, while β-diversity is used for depicting the variation in the identities and abundances of species among local assemblages (Mori et al., 2018). Another possible reason is that precipitation is not the only impact factor, other factors, including vegetation types (e.g., functional groups), temperature, grazing strategy, and topography may also affect the species diversity indices (Krner, 2003; Hodkinson, 2005; Wu et al., 2012; Li et al., 2020). For example, it was revealed that different functional groups of alpine grassland variably response to precipitation (Wu et al., 2014), and hence the diversity indices exhibited an obvious difference in the semiarid regions may be due to the differences in vegetation types (Figure 6; Supplementary Figure S1). In this study, we focused on revealing the potential effects of precipitation on the regulating species composition and tested the feasibility of UAV_B_ at multiple scales, the relative importance of impact factors (environmental and anthropogenic) that shape species diversity could be explored using the longer time-sequenced data of species diversity collected by FragMAP system (Zhang et al., 2021) at a large region in the future studies.

Usually, larger sampling areas mean higher representativeness, which will improve the estimation precision in high heterogeneity areas (Sun et al., 2018; Verrall et al., 2021). In this study, with the same data (13 sampling sites), we found a significant quadratic relationship between Pielou's *J* index investigated by UAV_B_ and precipitation, while there was a non-significant relationship between them based on the TSM_Q_ (Figure 5D). Similarly, the species richness and the Cody index estimated by UAV_B_ exhibited a significant quadratic relationship to precipitation, while it was a significant linear relationship estimated by TSM_Q_ (Figures 5A,F). Meanwhile, the other three diversity indices showed a similar trend (quadratic relationship) (Figures 5B,C,E). Hence, it is necessary and urgent to construct a standardized monitoring method (including the sampling area) to reveal the relationships between PSD and environmental gradients (Mori et al., 2018; Verrall et al., 2021). Especially, dynamic appraisal of diversity based on more samples is necessary for the heterogeneous distribution of species (Ge et al., 2018; Mori et al., 2018), for example, the 37 UAV-based samples covered different vegetation types in the semiarid regions (Figures 6, 7; Supplementary Figure S1, Table S1).

### Limitations of UAV_B_ and Future Work

We proposed and improved the UAV-based multiscale PSD monitoring method, and tested the method in the alpine grassland of SRYR on QTP. The method exhibited higher precision, efficiency, and representativeness (Figures 4–7). However, we do acknowledge that there is some room for further improvement. First, four species, i.e., *S. pumilus, A. serpyllifolia, E. arvense*, and *G. wilfordii*, failed to be identified on UAV photographs (Supplementary Table S1). However, this has a limited effect on the UAV_B_ method, for two reasons: (1) these species were rare, and their density, cover, and biomass were <1% based on the quadrats data of this study and former experiments (Sun et al., 2015, 2018); and (2) the Shannon index places more weight on rare species, while the Simpson index places more weight on abundant species (Klein et al., 2004). The significant linear relationships between estimated values of the two methods suggest that the errors caused by the rare species were limited in this study (*P* < 0.001; Figures 4B,C) and by the data collected from household pastures (Sun et al., 2018). While the precision of species identification still needs to be improved. Given that the species that failed to be identified are creeping or low-growing plants, some potential ways may be helpful to identify more species correctly. For example, improving the resolution of aerial photographs could identify more fine characteristics of each species at a fixed height. Furthermore, taking additional (e.g., ~10) aerial photographs manually at 0.5 m within the range of the Belt route after flying automatically will make it possible to identify species under the canopy, as the airflow made by the UAV's propellors and the higher ground resolution (because the lower height) may affect the quality of photographs and thus species recognition. Second, to date, species recognition depended on visual identification, requiring substantial time for aerial photographs analysis. Given the presence-based data of the UAV_B_ method, the plant species identification automatically by machine learning algorithm may further improve the UAV_B_'s efficiency (Lu and He, 2017; Sun et al., 2018).

Although the UAV_B_ method had been used for species composition monitoring in some specific areas, e.g., household pastures (Sun et al., 2018), Shule River Basin (Qin et al., 2020), and foreland of the Urumqi Glacier (Wei et al., 2021). It is still necessary to test the feasibility of different types of grassland, e.g., typical grassland and desert grasslands in arid and semi-arid areas. Meanwhile, it is also valuable to explore the applications of UAV_B_ at larger scales (the β- and γ-diversity), which is essential in the context of ecosystem multifunctionality but has received much less attention to date (Mori et al., 2018).

Recently, many researchers have recognized that the results of botanical studies are not consensus in plot experimental and natural communities (Mori et al., 2018; Genung et al., 2020). It is urgent to conduct long-term *in situ* studies (that is, the botanical studies in natural communities) to reveal the botanical characteristics on a large scale (Mori et al., 2018). Based on the FragMAP system, the sampling process could be separated into UAV-field samples and net-cooperation data extraction (Gao et al., 2020). UAV-field sampling has higher efficiency than field sampling (high-density sampling sites; Supplementary Figure S1), and net-cooperation data analysis at laboratories ensures accuracy (especially for identifying some rare species), which overcome the temporal and spatial limitations of data analysis of species diversity. Therefore, the UAV_B_ method based on the FragMAP system provides feasibility to study real-world natural communities at multiple scales, which may reconcile the contradictions from plot experimental- and natural-based studies.

## Conclusion

We demonstrate that UAV_B_ features with larger monitoring areas and more species, which was more representative of heterogeneous grassland. We show that weighing dominance of species may improve species diversity indices' precision based on aerial photographs of the FragMAP system. In SRYR, the species diversity increased and then tended to be stable with the increase of precipitation. Further studies are warranted (1) to reveal spatial and temporal pattern species composition and the impact factors, (2) to apply UAV_B_ in different types of grassland, and (3) to develop an automatic identification system of grassland plant species.

## Data Availability Statement

The original contributions presented in the study are included in the article/Supplementary Material, further inquiries can be directed to the corresponding author.

## Author Contributions

SY and YS: conceptualization, methodology, writing, reviewing, and editing. YS: software, data curation, and project administration. YY, YQ, and ZZ: validation. YY, ZZ, YL, WJ, QB, ML, and YS: investigation. YS, YL, WJ, QB, ZZ, and JW: writing the original draft preparation. ML and YQ: visualization. SY: supervision. All authors contributed to the article and approved the submitted version.

## Funding

This study was jointly supported by grants from the National Natural Science Foundation of China (31901393, 42071056) and the Natural Science Foundation of Jiangsu Province (BK20201439).

## Conflict of Interest

The authors declare that the research was conducted in the absence of any commercial or financial relationships that could be construed as a potential conflict of interest.

## Publisher's Note

All claims expressed in this article are solely those of the authors and do not necessarily represent those of their affiliated organizations, or those of the publisher, the editors and the reviewers. Any product that may be evaluated in this article, or claim that may be made by its manufacturer, is not guaranteed or endorsed by the publisher.
