# Supplementary material for: An Improved Method for Monitoring Multiscale Plant Species Diversity of Alpine Grassland Using UAV: A Case Study in the Source Region of the Yellow River, China
Source: Front Plant Sci. 2022 Jun 9;13:905715. doi: 10.3389/fpls.2022.905715 (PMC9218072; doi:10.3389/fpls.2022.905715)
Supplement: Supplementary file 1 [file Data_Sheet_1.docx]

***Supporting information***

**Table S1.** List of recorded species in four typical alpine grasslands of the headwater region of the Huanghe River by UAVs-based method and traditional quadrat method (103 species in all). Growth form (GF) and life cycle (LC) are listed for all species. Growth forms: grass/sedge (grass), forb (forb), shrub (shrub). Life cycles: annual (ann), biennial (bie), and perennial (per).

| Species | GF | LC | Species | GF | LC |
| --- | --- | --- | --- | --- | --- |
| *Elymus nutans* | grass | per | *Saussurea pachyneura* | forb | per |
| *Poa poophagorum* | grass | per | *Cremanthodium lineare* | forb | per |
| *Poa chalarantha* | grass | per | *Artemisia hedinii* | forb | ann |
| *Koeleria cristata* | grass | per | *Saussurea nigrescens* | forb | per |
| *Stipa aliena* | grass | per | *Heteropappus bowerii* | forb | per |
| *Stipa purpurea* | grass | per | *Cirsium esculentum* † | forb | Per |
| *Achnatherum splendens* | grass | per | *Saussurea arenaria* | forb | Per |
| *Kobresia graminifolia* | grass | per | *Saussurea stoliczkae* ††† | forb | Per |
| *Kobresia tibetica* | grass | per | *Potentilla fragarioides* | forb | Per |
| *Kobresia robusta* ††† | grass | per | *Potentilla bifurca* | forb | Per |
| *Kobresia pygmaea* | grass | per | *Potentilla anserine* | forb | Per |
| *Scirpus pumilus* †† | grass | per | *Sanguisorba filiformis* | forb | Per |
| *Carex atrofuscoides* | grass | per | [*Sibbaldia procumbens*](http://www.baidu.com/link?url=dlBu5KYJEivIOCbmiaRkDtoVpqtonOKfj7GsxB98GBt3grMm7GIsi8UROoLegRj3m_rAVJxNTkr6I5F-6fO6xq) | forb | Per |
| *Carex orbicularis* | grass | per | *Potentilla acaulis* | forb | Per |
| *Blysmus sinocompressus* † | grass | per | *Potentilla fruticosa* | Shrub | Per |
| *Anemone rioularis* | forb | per | *Pleurospermum pulszkyi* | forb | Per |
| *Anemone obtusiloba* | forb | per | *Carum carvi* | forb | Per |
| *Anemone trullifolia* | forb | per | *Heracleum millefolium* | forb | Per |
| *Trollius farreri* | forb | per | *Angelica nitida* † | forb | Per |
| *Delphinium grandiflorum* | forb | per | *Arenaria serpyllifolia* †† | forb | ann or bie |
| *Ranunculus tanguticus* | forb | per | *Cerastium arvense* | forb | Per |
| *Thalicturn alpinum* | forb | per | *Stellaria media* | forb | ann or bie |
| *Caltha palustris* | forb | per | *Arenaria kansuensis* | forb | Per |
| *Aconitum carmichaeli* | forb | per | *Equisetum arvense* †† | forb | Per |
| *Oxytropis kansuensis* | forb | per | *Plantago depressa* | forb | ann or bie |
| *Oxytropis caerulea* ††† | forb | per | *Polygonum viviparum* | forb | Per |
| *Gueldenstaedtia diversifolia* | forb | per | *Polygonum sibiricum* | forb | Per |
| *Astragalus polycladus* | forb | per | *Rumex patientia* | forb | Per |
| *Thermopsis lanceolata* | forb | per | *Rheum spiciforme* | forb | Per |
| *Caragana tangutica* | forb | per | *Geranium wilfordii* †† | forb | Per |
| *Oxytropis falcata* | forb | per | *Allium sikkimense* | forb | Per |
| *Pedicularis verticillate* † | forb | ann | *Fritillaria cirrhosa* † | forb | Per |
| *Pedicularis cranolopha* | forb | per | *Euphorbia micractina* | forb | Per |
| *Pedicularis chinensis* | forb | ann | *Lamiophlomis rotata* | forb | Per |
| *Euphrasia regelii* | forb | ann | *Ajuga lupulina* † | forb | Per |
| *Lancea tibetica* | forb | per | *Dracocephalum heterophyllum* † | forb | Per |
| *Lagotis brachystachya* | forb | per | *Elsholtzia ciliata* | forb | Per |
| *Veronica eriogyne* | forb | ann or bie | *Stellera chamaejasme* | forb | Per |
| *Halenia elliptica* | forb | ann | *Saxifraga stolonifera* | forb | Per |
| *Gentiana spathulifolia* | forb | ann | *Parnassia trinervis* | forb | Per |
| *Gentiana sino-ornata* | forb | per | *Lepidium apetalum* | forb | Per |
| *Gentiana macrophylla* | forb | per | *Nardostachys chinensis* | forb | Per |
| *Gentianopsis barbata* | forb | ann or bie | *Cardamine tangutorum* | forb | Per |
| *Swertia bimaculata* | forb | ann | *Descurainia sophia* | forb | Ann |
| *Aster flaccidus* † | forb | per | *Morina kokonorica* | forb | Per |
| *Aster alpinus* | forb | per | *Galium verum* | forb | Per |
| *Aster himalaicus* ††† | forb | per | *Przewalskia tangutica* † | forb | Per |
| *Saussurea hieracoides* | forb | per | *Chenopodium album* | forb | Ann |
| *Saussurea stella* | forb | bie | *Androsace gmelinii* | forb | Ann |
| *Anaphalis lacteal* | forb | per | *Pomatosace filicula* | forb | ann or bie |
| *Leontopodium souliei* | forb | per | *Androsace tapete* ††† | forb | Per |
| *Artemisia sieversiana* | forb | ann or bie | *Glaux maritima* | forb | Per |
| *Ajania tenuifolia* † | forb | per | *Microula tibetica* ††† | forb | Per |
| *Ligularia virgaurea* | forb | per | *Myricaria prostrata* ††† | forb | Per |
| *Ligularia sagitta* † | forb | per | *Rhodiola rosea* ††† | forb | Per |
| *Taraxacum mongolicum* | forb | per |  |  |  |

† and †† mean the species were not identify by the traditional quadrat method (11species) and UAVs-based method (4 species) within 13 sampling sites, respectively; ††† means the species (8 species) were identify within the 24 sampling sites (excepting the 13 sampling sites) by the UAVs-based method.

**Table S2.** All the potential possible cases of weighing species dominance (Rank based on species dominance that were estimated by coverage on the aerial photographs).

| Possible cases | Rank1 species | | Rank2 species | | Rank3 species | | |
| --- | --- | --- | --- | --- | --- | --- | --- |
|  | ① | ② | ① | ② | ① | … |  |
| I |  |  |  |  | all 1 | | |
| II | 3 |  |  |  |  |  |  |
| III | 3 |  | 2 |  |  |  |  |
| Ⅳ | 3 |  | 2 | 2 |  |  |  |
| Ⅴ | 3 | 3 |  |  |  |  |  |
| Ⅵ | 3 | 3 | 2 |  |  |  |  |
| Ⅶ | 3 | 3 | 2 | 2 |  |  |  |


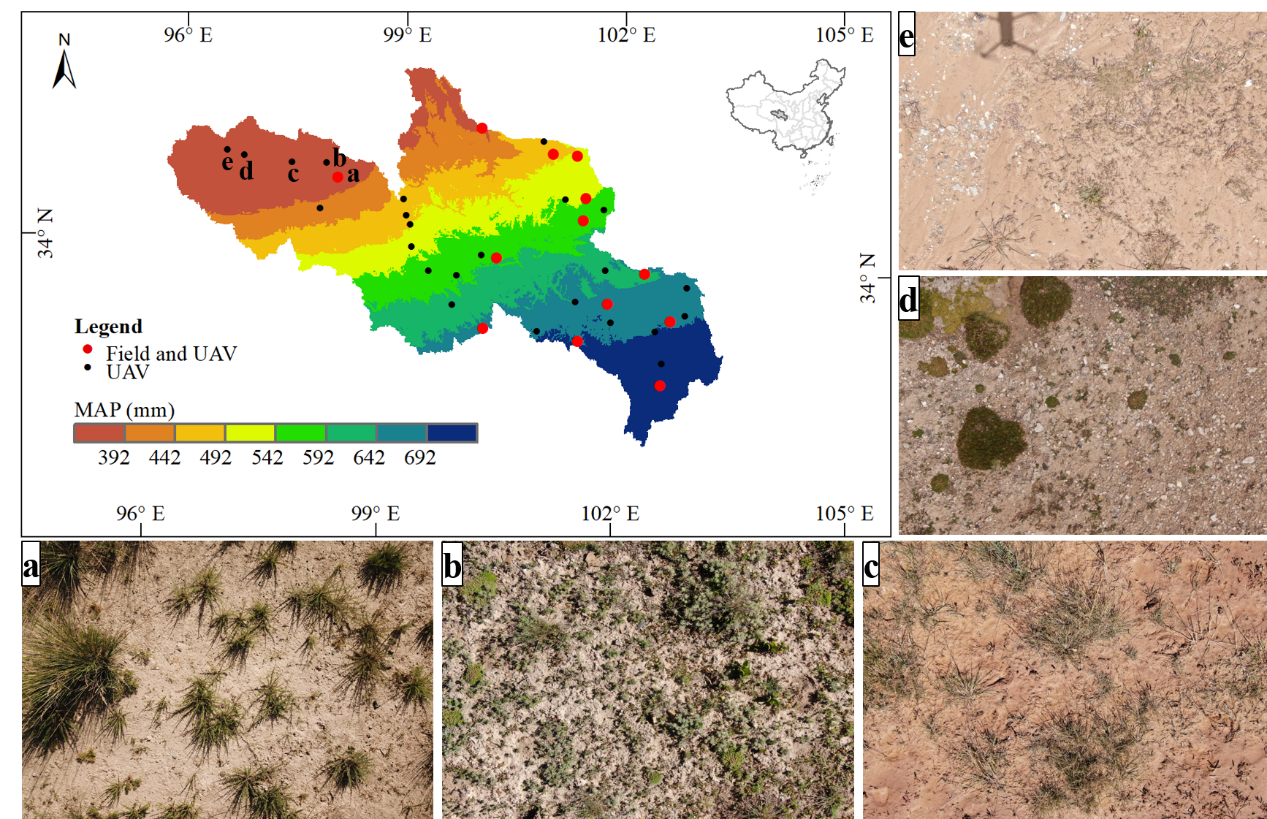


**Figure S1** The typical aerial photographs (different community assembly) taken at 5 sampling sites set in the semiarid region of source region of the Yellow River.
